# Intramuscular Ricin Poisoning of Mice Leads to Widespread Damage in the Heart, Spleen, and Bone Marrow

**DOI:** 10.3390/toxins11060344

**Published:** 2019-06-16

**Authors:** Anita Sapoznikov, Amir Rosner, Reut Falach, Yoav Gal, Moshe Aftalion, Yentl Evgy, Ofir Israeli, Tamar Sabo, Chanoch Kronman

**Affiliations:** 1 Department of Biochemistry and Molecular Genetics, Israel Institute for Biological Research, Ness-Ziona 76100, Israel; anitas@iibr.gov.il (A.S.); reutf@iibr.gov.il (R.F.); yoavg@iibr.gov.il (Y.G.); moshea@iibr.gov.il (M.A.); yentle@iibr.gov.il (Y.E.); ofiri@iibr.gov.il (O.I.); chanochk@iibr.gov.il (C.K.); 2 Veterinary Center for Preclinical Research, Israel Institute for Biological Research, Ness-Ziona 76100, Israel; amirr@iibr.gov.il

**Keywords:** ricin, intramuscular, heart, cardiomyocytes, spleen, bone marrow, thrombocytopenia, coagulopathy, hemorrhages

## Abstract

Ricin, a lethal toxin derived from castor oil beans, is a potential bio-threat due to its high availability and simplicity of preparation. Ricin is prepared according to simple recipes available on the internet, and was recently considered in terrorist, suicide, or homicide attempts involving the parenteral route of exposure. In-depth study of the morbidity developing from parenteral ricin poisoning is mandatory for tailoring appropriate therapeutic measures to mitigate ricin toxicity in such instances. The present study applies various biochemical, hematological, histopathological, molecular, and functional approaches to broadly investigate the systemic effects of parenteral intoxication by a lethal dose of ricin in a murine model. Along with prompt coagulopathy, multi-organ hemorrhages, and thrombocytopenia, ricin induced profound morpho-pathological and functional damage in the spleen, bone marrow, and cardiovascular system. In the heart, diffuse hemorrhages, myocyte necrosis, collagen deposition, and induction in fibrinogen were observed. Severe functional impairment was manifested by marked thickening of the left ventricular wall, decreased ventricular volume, and a significant reduction in stroke volume and cardiac output. Unexpectedly, the differential severity of the ricin-induced damage did not correlate with the respective ricin-dependent catalytic activity measured in the various organs. These findings emphasize the complexity of ricin toxicity and stress the importance of developing novel therapeutic strategies that will combine not only anti-ricin specific therapy, but also will target ricin-induced indirect disturbances.

## 1. Introduction

Ricin, a highly toxic protein isolated from the castor plant *Ricinus communis*, exerts its noxious effect by site-specific depurination of ribosomal 28S rRNA, which in turn leads to cessation of protein synthesis and cell death. Ricin is considered a bioterror threat of concern due to its ease of preparation, long-term stability, and worldwide availability in large quantities. Exposure to ricin can occur by oral ingestion, inhalation, or parenteral administration, though the features of poisoning and severity of toxicity vary markedly with the route of exposure. The most relevant routes of exposure to ricin in military or terrorist scenarios are inhalation and parenteral administration. Whichever scenario is considered, early diagnosis of ricin poisoning is necessary in order to take therapeutic steps to mitigate the toxicity. The consequences of pulmonary ricinosis have been extensively investigated by our group [1,2] and by others [3,4,5,6,7,8,9,10]. In contrast, ricin poisoning by parenteral route, i.e., intramuscularly penetration, has not yet been well-defined. One of the few examples of parenteral ricin poisoning is that of Georgi Markov, a Bulgarian journalist who was assassinated in 1978 with a ricin-laden umbrella tip, which was used to stab his right thigh [11]. This case, though taking place several decades ago, is exceptional and very enlightening, as it has been comprehensively reviewed and provided a rudimentary chronological timeline for the clinical manifestations following parenteral ricin exposure in humans. Markov, who was injected with approximately 500 µg toxin, experienced immediate pain at the site of injection and the first symptoms developed over the next 24 h. At admission to a hospital approximately 35 h after the incident, local necrotic lymphadenopathy, elevated white cell counts, and a 6 cm diameter circular inflamed region of induration at the site of umbrella tip insert were documented. Two days after the attack, renal failure, hypotension, tachycardia, and hypothermia were apparent. Electrocardiographic monitoring on day 3 suggested complete atrioventricular conduction block, which advanced to cardiac arrest that occurred in the morning of this day. The autopsy showed, in addition to the aforementioned clinical manifestations, pulmonary edema, necrosis in the small intestine, and interstitial hemorrhages throughout the heart muscle and other organs. Since the Markov incident, several suicide and homicide cases have allegedly involved the use of ricin [12,13,14,15,16]. In addition, in recent years, ricin has become a tool of choice in bioterrorism, assumedly due to the broad dissemination of ricin production methods through internet tutorials. In mid-2018, German security services uncovered a terrorist plot involving the use of an improvised explosive device loaded with ricin [17]. This case, illustrating the existing threat of bioterrorism with weaponized ricin, joins other reported cases of extremist attempts in England, France, and the United States [18,19]. In cases of sharp or explosive devices, ricin would be expected to penetrate the skin, and then enter the muscle and blood stream, thereby involving a mixture of subcutaneous, intramuscular, and intravascular exposures.

In the present study, we employed diverse biochemical, hematological, histopathological, molecular, and functional approaches to chart the developing morbidity following exposure of mice to a lethal dose of ricin by the intramuscular route. The magnitude of damage inflicted to various organs of the ricin-intoxicated mice differed widely and did not correlate with the ricin-dependent catalytic activity measured in the corresponding organs. These findings suggest that at least some of the severe pathological manifestations of intramuscular exposure to ricin are an indirect outcome of ricin catalytic activity. 

## 2. Results

### 2.1. Biochemical and Hematological Changes in Mice Following Exposure to Ricin

To characterize the morbidity induced by parenteral exposure to ricin, mice were intramuscularly exposed to a lethal dose of the toxin (2LD_50_, 18 µg/kg body weight) and various physiological parameters were examined. Body temperature was not affected during the first 24 h post-exposure, yet dropped markedly from 37.7 ± 0.8 °C (temperature measured before exposure) to 31.7 ± 2.1 °C and 29.5 ± 3.3 °C at 48 and 72 h post-exposure, respectively (Table 1). Reduced glucose levels, discerned already at 24 h, eventually dropped from 228 ± 48 mg/dL in naive mice to 89 ± 33 mg/dL at 72 h post-exposure (Table 1). Hematological analysis of blood samples collected from ricin-intoxicated mice revealed an increase over time in white blood cell (WBC) counts in general and in neutrophil counts in particular. Neutrophil counts in the circulation at 48–72 h post-exposure were ~6-fold higher than baseline (Table 1). Examination of coagulation rates in plasma samples collected at various time-points revealed a continuous prolongation in prothrombin time (PT) and activated partial thromboplastin time (APTT), and reduced coagulation performance, being evident already at 24 h post-exposure. Plasma fibrinogen levels were also elevated at 24 h post-exposure and continued to ascend at later time points (Table 1). In addition to altering plasma clotting-capacity, ricin intoxication reduced the levels of another crucial hemostatic component—the platelets. Ricin induced severe thrombocytopenia, with platelet counts displaying 4- and 5-fold decrease at 48 and 72 h post-exposure, respectively (Table 1). No changes in other blood cell populations, blood hemoglobin, or D-Dimer levels were noted (Table S1). 

Since coagulation and inflammation are interdependent systems, we further examined the pro-inflammatory response in mice. Elevated levels of interleukin 6 (IL-6) and tumor necrosis factor alpha (TNF-α) were detected at 24 h post-exposure and continued to rise until 48 h post-exposure, after which TNF-α levels descended at 72 h following intoxication, while IL-6 levels did not change. Since individual ricin-intoxicated mice displayed great differences in IL-1β levels, statistically significant alterations in the blood level of this cytokine could be measured only at 72 h post-exposure (Table 1). 

To evaluate the effect of ricin on organ functional integrity, serum biomarkers were measured. Alanine transaminase (ALT) and alkaline phosphatase (ALP), indicative for liver damage, were significantly elevated at 48 and 72 h post-exposure, respectively. Amylase (AMY), a marker for pancreatic function, displayed a ~2-fold increase starting from 48 h post-exposure. Blood urea nitrogen (BUN) and phosphorus (PHOS), biomarkers of renal damage, also showed an increase at 48 and 72 h post-exposure. Decreased levels of the electrolytes, Na^+^, and Cl^−^ were measured following 48 h post-exposure, while K^+^ levels were elevated at 72 h post-exposure (Table 1). Other parameters, such as albumin (ALB), bilirubin (TBIL), creatinine (CRE), total protein (TP), and globulin (GLOB), showed no change (Table S1).

Altogether, the data indicate that following intramuscular exposure to ricin, mice developed hypothermia, hypoglycemia, thrombocytopenia, along with coagulopathy, an elevated pro-inflammatory response, and mild abnormalities in liver, pancreatic, and renal function. 

### 2.2. Histopathological Alterations in Mice Following Exposure to Ricin

**Figure 1:**
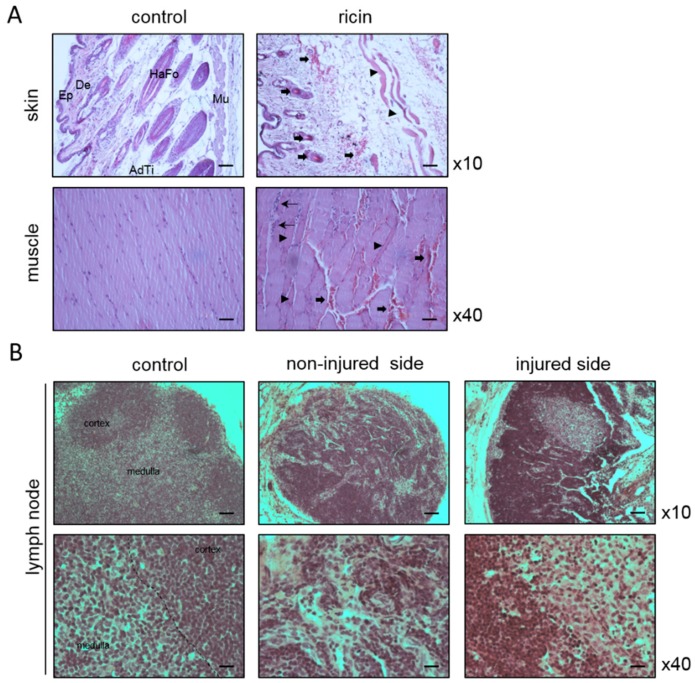
Histological analysis of the site of injury. Representative sections of skin and muscle from the right hip (**A**) and lymph nodes (LNs) (**B**) from non-intoxicated (control) and ricin intoxicated mice (2LD_50_, 18 µg ricin/kg body weight) at 96 h post-exposure. Thick arrows indicate hemorrhagic areas, characterized by erythrocyte extravasation; thin arrows indicate neutrophil infiltration; arrowheads indicate muscular damage. Ep = epidermis; De = dermis; HaFo = hair follicle; AdTi = adipose tissue; Mu = muscle. The sections were stained with hematoxylin and eosin (H&E), and images were captured under ×10 or ×40 magnification. Scale bars: 100 µm; *n* = 5 in each group.

Hemorrhagic areas, observed in the skin in close proximity to the site of the toxin injection, were characterized by erythrocyte extravasation into the tissue and the hair follicles. Muscular damage was observed as well. Degeneration of striated muscle fibers of the thigh was associated with hemorrhagic areas, neutrophil infiltration, and edema (Figure 1A). The ricin-induced damage of the skin and muscles was, however, of a local nature and was confined to the immediate vicinity of the injection site. Extensive damage of the inguinal lymph nodes (ILNs) was displayed in the injected as well as the non-injected hind limbs. Massive necrosis of the cells of the cortex and medulla rendered differentiation between the two compartments nearly impossible. Analysis of other LNs, such as axillary LNs, demonstrated several small necrotic areas in an otherwise unaffected structure (Figure 1B). 

**Figure 2:**
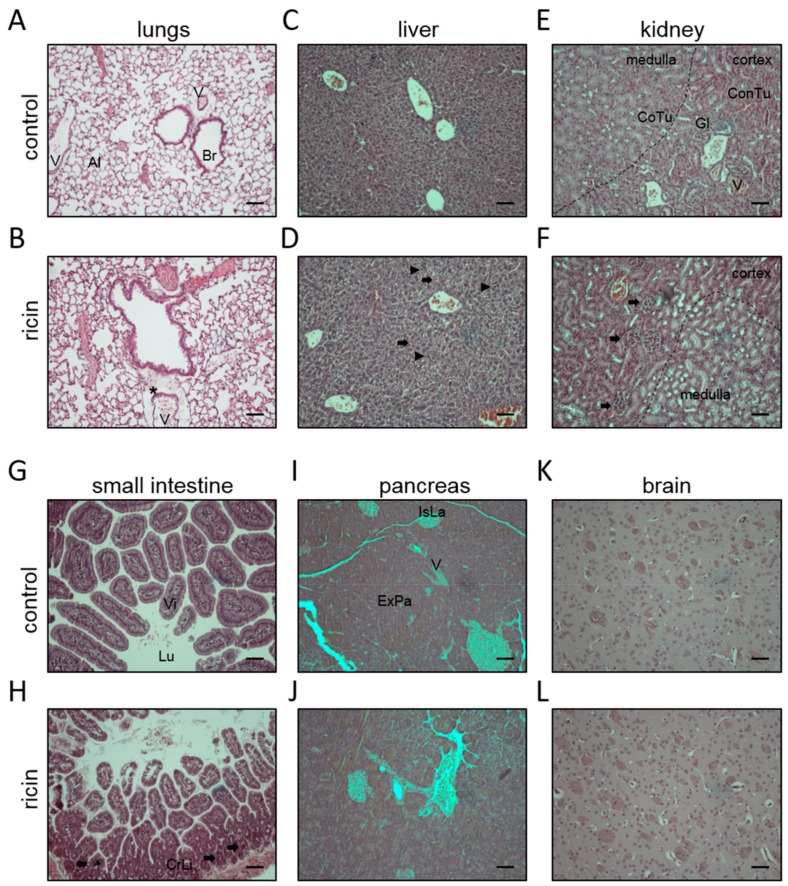
Organs of mice displaying minor damage following exposure to ricin. Representative sections of lungs (**A**,**B**), liver (**C**,**D**), kidney (**E**,**F**), small intestine (**G**,**H**), pancreas (**I**,J), and brain (**K**,**L**) from non-intoxicated (control) and ricin intoxicated (ricin, 2LD_50_, 18 µg ricin/kg body weight) mice at 96 h post-exposure. Thick arrows indicate hemorrhagic areas; arrowheads indicate focal necrosis; asterisks indicate edema. Br = bronchiole; V = blood vessel; Al = alveoli; Gl = glomerulus; ConTu = convoluted tubules; CoTu = collecting tubules; Vi = villi; Lu = lumen; CrLi = crypts of Lieberkuhn; IsLa = islets of Langerhans; ExPa = exocrine pancreas. The sections were stained with H&E, and images were captured under ×10 magnification. Scale bars: 100 µm; *n* = 5 in each group.

Histological analysis of the lungs demonstrated congestion of blood vessels, poor organization of the bronchiolar epithelia, edema, and to some extent, cell infiltration (Figure 2A,B). Focal hemorrhages and necrotic sites were evidenced in the liver, however, the overall structure of this organ remained preserved (Figure 2C,D). Alterations in the kidney were discerned mainly in the cortex region, with some hemorrhages in the glomerulus, but as in case of the liver, no substantial changes in the organ structure were observed (Figure 2E,F). In the small intestine, the structure of the villi remained intact, however, hemorrhages in the base of the villi, the crypts of Lieberkuhn, were noticed (Figure 2G,H). In the pancreas (Figure 2I,J) and the brain (Figure 2K,L), we did not detect any aberrant morphology. 

**Figure 3:**
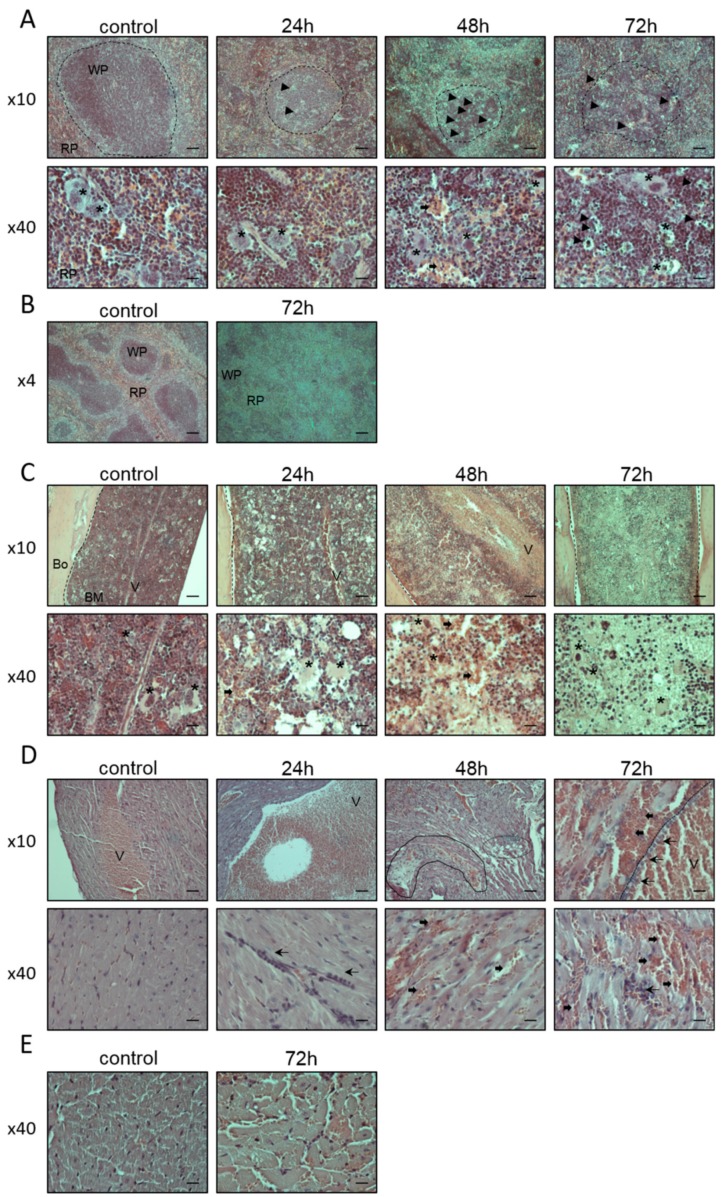
Ricin-induced damage in the spleen, bone marrow (BM), and heart of intoxicated mice. Representative sections of spleen (**A**,**B**), BM (**C**), and longitudinal-sections (**D**) and cross-sections (**E**) of the heart, from non-intoxicated (control) and ricin intoxicated (2LD_50_, 18 µg ricin/kg body weight) mice at 24, 48 and 72 h post-exposure. Thick arrows indicate hemorrhagic areas; thin arrows indicate neutrophil infiltrates; arrowheads indicate cellular atrophy; asterisks indicate megakaryocytes. White pulps in the spleen sections are delineated by dashed lines (**B**). In the BM sections, dashed lines separate bone (left) and marrow (right) (**C**). Vacuolization in the heart section is delineated by dashed oval structures and muscle atrophy is delineated by solid line structures; dashed line separates between muscle (left) and blood vessel (right) (**D**). Wp = white pulp; Rp = red pulp; V = blood vessel; Bo = bone; BM = bone marrow. The sections were stained with H&E, and images were captured under ×4, ×10, and ×40 magnification. Scale bars: 100 µm; *n* = 5 in each group.

In contrast to the above-mentioned tissues, which exhibited small-scale lesions, widespread damage was observed in the spleen, which exhibited atrophy of the white pulps, mainly at 48 and 72 h after intoxication (Figure 3A,B). Decreased cellularity was evident also in the red pulps. Higher magnification allowed detection of focal necrosis, hemorrhages, and gradual pyknosis of megakaryocytes (Figure 3A). In parallel to the spleen, substantial damage, including blood vessel congestion, hemorrhages, death of megakaryocytes, and tissue atrophy, was detected also in the BM (Figure 3C). These ricin-induced alterations were emphasized with the passage of time after intoxication. Insult of the marrow was not confined to the limb into which ricin was injected, but rather occurred systemically. Interestingly, besides the lymphoid organs (spleen, BM), substantial damage was detected in the heart of the mice. Neutrophil infiltration to the heart was noted already at 24 h after exposure. At 48 and 72 h post-exposure, diffuse hemorrhages and neutrophilic infiltrates between muscle strands, myocyte disconnection and loss of striation, atrophic areas, focal vacuolization, and interstitial edema were identified (Figure 3D). Myocyte enlargement was documented in the cross-sections of the heart (Figure 3E). 

**Figure 4:**
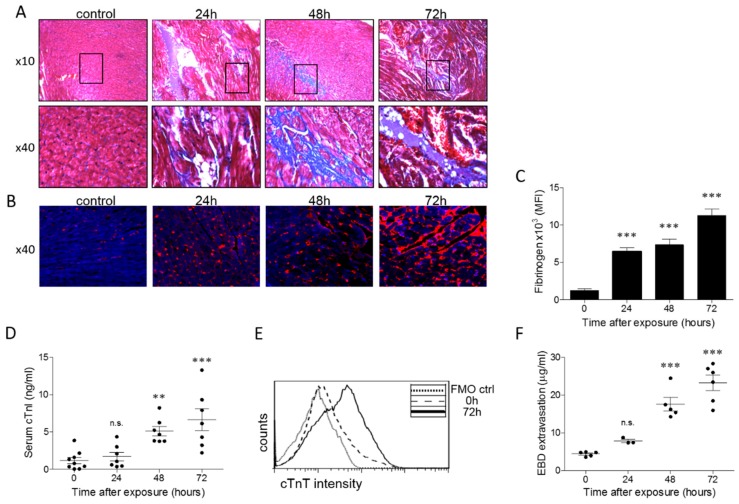
Remodeling of the heart in ricin-intoxicated mice. (**A**) Collagen deposition was determined by Masson’s-Trichrome staining in hearts removed from ricin-intoxicated mice (2LD_50_, 18 µg ricin/kg body weight), at the indicated post-exposure time-points. Hearts of non-intoxicated mice serve as control; *n* = 5 in each group. (**B**) Confocal immunofluorescence (red) shows expression of fibrinogen in the heart of non-intoxicated mice versus ricin-intoxicated mice (blue, diamidino-2-phenylindole-6,’4 (DAPI) staining of nuclei); *n* = 3 (control, and 24 h), and *n* = 5 (48 and 72 h). (**C**) Bars represent the immunofluorescence staining intensity of fibrinogen expressed as mean fluorescence intensity (MFI). (**D**) The cTnI levels were determined in serum samples removed from non-intoxicated mice (0 h) and from ricin-intoxicated mice at the indicated time-points; *n* = 9 (control) and *n* = 7 (24–72 h). (**E**) Intracellular levels of cTnT in cardiomyocytes in non-intoxicated mice (0 h) versus 72 h after ricin intoxication; fluorescent minus one control (FMO), cardiomyocytes that were not stained for cTnT; *n* = 3 (control and 24 h) and *n* = 4 (48 and 72 h). (**F**) Heart EBD following ricin intoxication. Control or ricin-intoxicated mice were intravenously injected with 50 mg/kg EBD at the indicated time points and hearts harvested 1 h later were monitored for EBD content; *n* = 5, 3, 5, and 6 for 0, 24, 48, and 72 h, respectively; * *p* < 0.05, ** *p* < 0.01, *** *p* < 0.001 in comparison to non-intoxicated mice.

Focal fibrosis, observed in the heart as early as 24 h after exposure, further increased at 48–72 h post-exposure (Figure 4A). Elevated levels of fibrinogen, an acute phase reactant that can rise in cases of inflammation, myocardial damage, or bleeding, were detected in the hearts of the ricin-intoxicated mice throughout the time-span of the experiment (Figure 4B,C). Cardiac troponin I (cTnI) and cardiac troponin T (cTnT), components of the contractile regulatory protein complex that serve as markers for myocardial injury and necrosis, were measured as well. Serum levels of cTnI, elevated at 48 h post-exposure, continued to increase at 72 h after exposure (Figure 4D), while cTnT staining of myocardiocytes showed high intracellular levels of this protein (Figure 4E). Hyperpermeability of the myocardium, as tested by the Evans Blue dye (EBD) extravasation assay, was evident at 48–72 h after ricin intoxication (Figure 4F). Altogether, this data is indicative of profound injury of the heart. 

### 2.3. Functional Alterations in the Myocardium Following Intramuscular Ricin Intoxication

**Figure 5:**
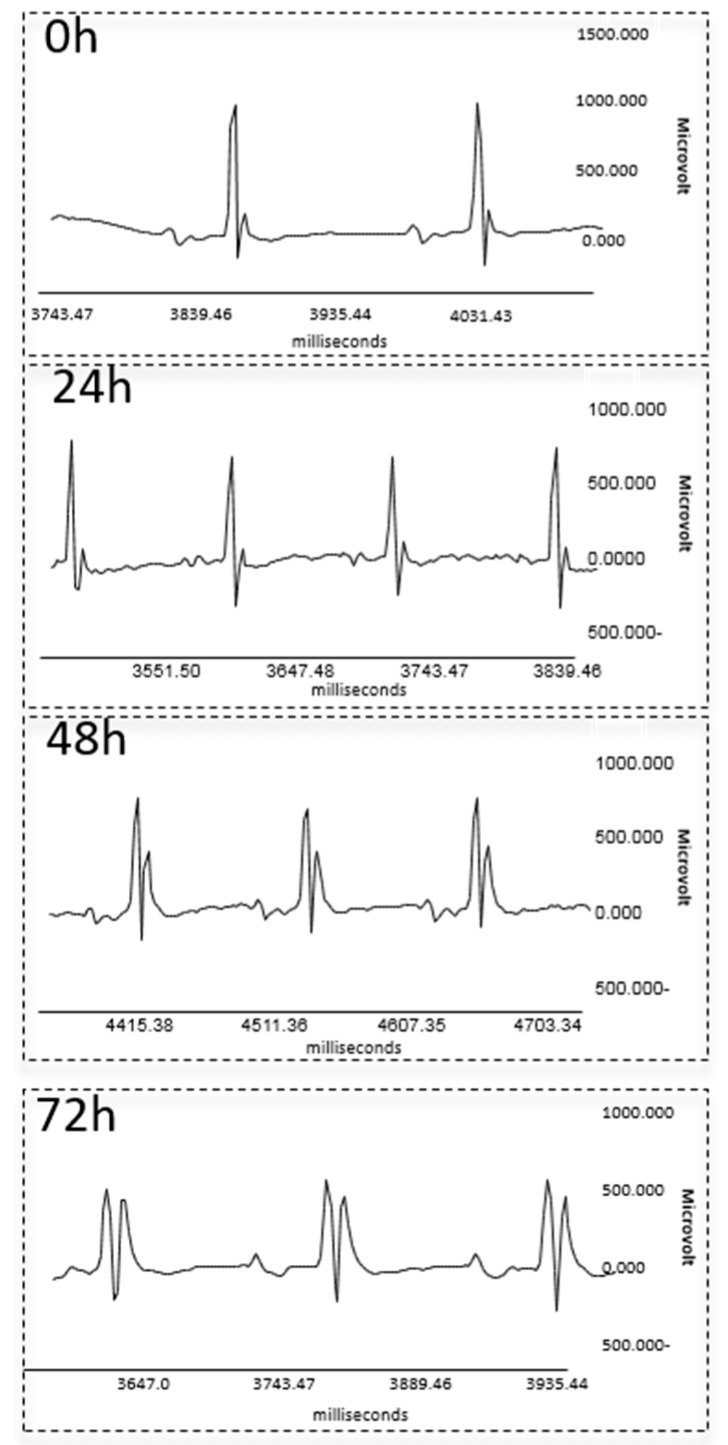
Alterations in ECG parameters in ricin-intoxicated mice. ECG measurements were performed immediately before i.m. exposure to 2LD_50_ ricin (18 µg ricin/kg body weight) or at the indicated time points thereafter. A representative ECG before and after exposure to ricin.

Electrocardiographic analyses (ECG) were performed to determine whether the prominent histopathological alterations observed in the heart are reflected in myocardial dysfunction. Transient increase in the heart rate at 24 h, as well as a prominent elevation of the J wave amplitude at 48 and 72 h after ricin exposure, were detected in a representative ECG image by unaided eye (Figure 5). A more detailed and quantitative analysis of the ECG records confirmed a temporary elevation in heart rate from 425 ± 70 bpm in naïve mice to 506 ± 50 bpm at 24 h after toxin exposure. Later, at 72 h post-exposure, heart rate decreased to 357 ± 67 bpm. Additional early changes observed at 24 h after toxin administration were a decrease in R wave amplitude and increase in J wave amplitude. In addition to the aforementioned changes, we noted at 48 h post-exposure, a prolongation of the P wave duration (P segment), which serves as a measure of intra-atrial conduction, and a widening of the QRST interval, an indication of prolonged duration of ventricular depolarization and repolarization. QRST increased significantly as a result of widening of the J wave. At 72 h post-exposure, extensions of the PQ (atrioventricular conduction) and QRS intervals, the latter indicating a prolongation of ventricular conduction time, were documented. The changes observed at earlier time-points, namely, increase in J wave amplitude, decrease in R wave amplitude, and the prolongations of the P segment and QRST interval, were even more pronounced at this late time point (Table 2). 

**Figure 6:**
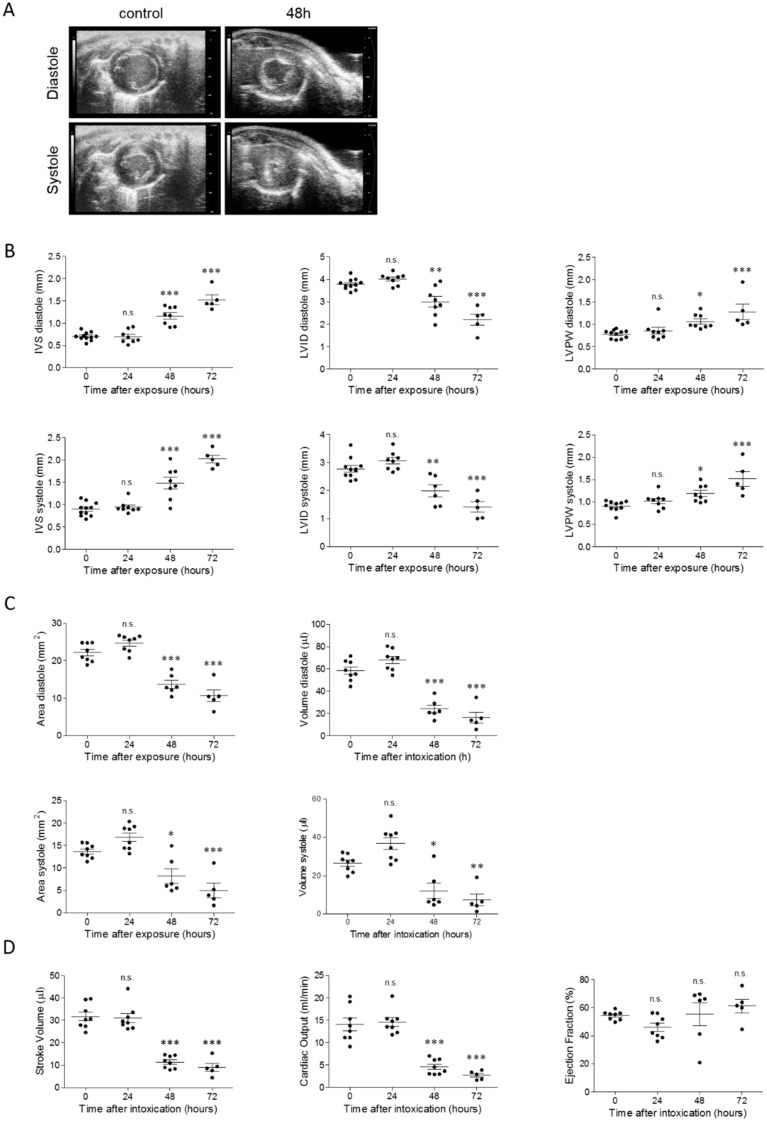
Structural and hemodynamic alterations in hearts of ricin-intoxicated mice. (**A**) Representative echocardiographic images of short-axis view of the LV of non-intoxicated or 48 h after ricin-intoxicated mice (2LD_50_, 18 µg ricin/kg body weight) in diastolic and systolic state. (**B**) LV diastolic and systolic dimensions. IVS = interventricular septum; LVID = left ventricular interior diameter; LVPW = left ventricular posterior wall. (**C**) LV area and volume before and at indicated time points after exposure to ricin. (**D**) Hemodynamic parameters: stroke volume, cardiac output, and ejection fraction before and at indicated time points after exposure to ricin; *n* = 5–10; * *p* < 0.05, ** *p* < 0.01, *** *p* < 0.001 in comparison to non-intoxicated mice.

Further assessment of the cardiac performance in ricin-intoxicated mice was achieved by transthoracic electrocardiography of the left ventricle (LV). First, we evaluated cardiac structure by analysis of the LV in a short-axis view during diastole and systole. Comparison between the ventricle images of ricin-intoxicated and naïve mice at the level of the papillary muscles revealed significant narrowing of the chamber dimensions, thickening of the ventricular wall, and irregular shaping of the endocardium of the intoxicated mice, both in diastolic and systolic state (Figure 6A). By tracing of the short-axis cineloops we detected a substantial increase in the thickness of the interventricular septum (IVS) and LV posterior wall (LVPW) during relaxation and contraction at 48–72 h after exposure to ricin. Concomitantly, the LV interior diameter (LVID) was significantly reduced at the same time points (Figure 6B). Observation by the parasternal long-axis view revealed diminished diastolic and systolic area and volume of the LV chamber at 48 h, and even more so at 72 h after exposure (Figure 6C). Functional parameters were assessed by use of the LV-trace tool (Figure 6D). The amount of the blood pumped out of the heart during a single heartbeat—the stroke volume—was dramatically decreased at 48–72 h after toxin administration. As an inevitable consequence of the reduction in heart rate (Table 2) and stroke volume, reduction in cardiac output (the amount of blood pumped by LV per minute) at these time points was recorded as well. Moreover, since the stroke volume and the end-diastolic volume in the ricin-intoxicated mice were reduced in a proportional manner, the ejection fraction, measuring the efficiency of heart contraction, and a quotient between these two measurements remained unaltered. 

### 2.4. Cellular Alterations in the Spleen, Bone Marrow, and Heart of Ricin-Intoxicated Mice

**Figure 7:**
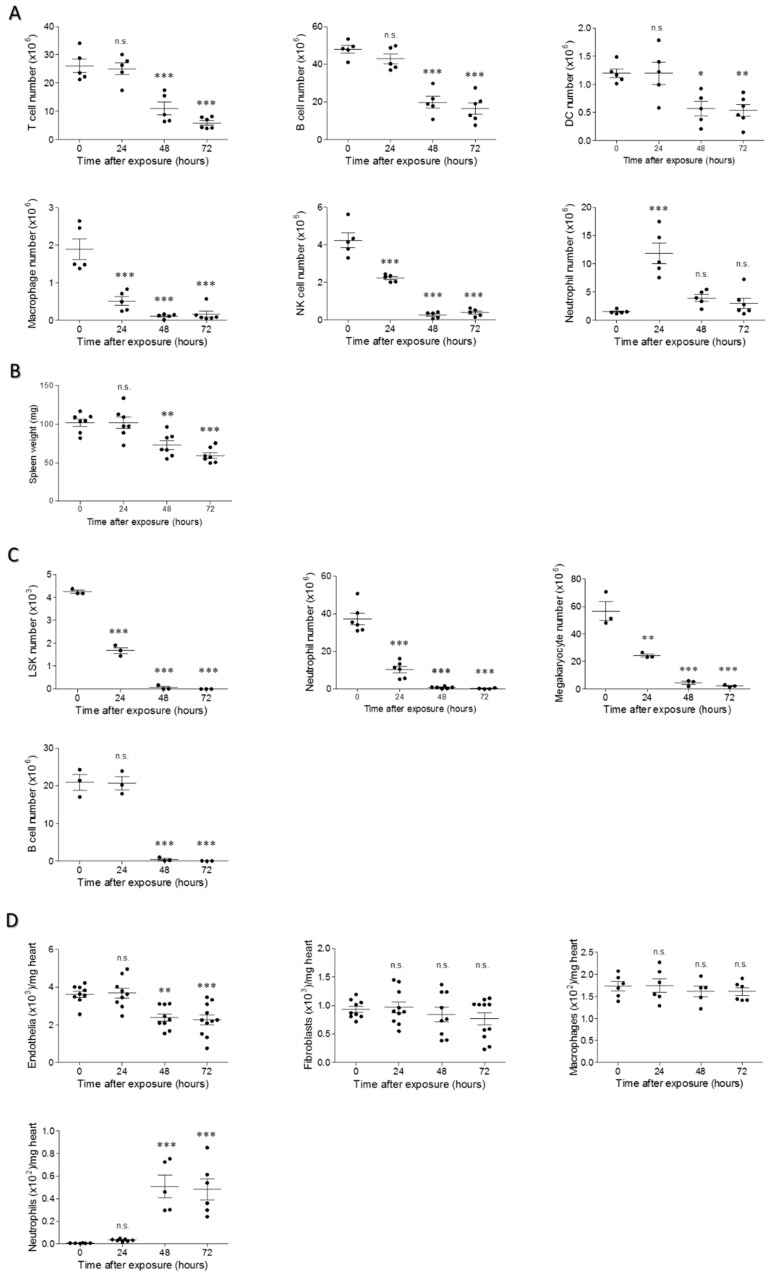
Cell counts at different time points following ricin intoxication. (**A**) Total spleen, (**C**) two femurs and two tibia, and (**D**) heart cells, isolated from intramuscularly ricin-intoxicated (2LD_50_, 18 µg ricin/kg body weight) mice at the indicated post-exposure time-points were stained for cell type specific surface markers and analyzed by flow cytometry. (**B**) Weights of spleens collected from intramuscularly ricin-intoxicated mice at the indicated post-exposure time-points. Splenic cells, *n* = 5; spleen weight, *n* = 7; BM cells, *n* = 3–6; heart cells, *n* = 5–11; * *p* < 0.05, ** *p* < 0.01, *** *p* < 0.001 in comparison to non-intoxicated mice.

It has been previously shown in the mouse ricin pulmonary exposure model that the various cell types populating the lungs are differentially susceptible to toxin binding and ricin-induced protein synthesis arrest and death. Thus, alveolar macrophages and dendritic cells are the first cell types to be affected by the toxin, B cells, endothelial, and alveolar epithelial cells are partially eliminated from the lung at later time-points, while neutrophils are virtually refractive to ricin-mediated elimination [6,20,21,22]. To better understand the pathology of intramuscular ricinosis, we performed a quantitative analysis of differential cell populations in the most affected organs, spleen, BM, and heart. In the spleen, a 2- and 5-fold reduction in T lymphocytes and a 2- and 3-fold reduction in B lymphocytes were measured at 48 and 72 h post-exposure, respectively. Likewise, a 2-fold reduction in the dendritic cell population could be discerned at 48 and 72 h after intoxication. Reduction by at least one order of magnitude was observed in splenic macrophages and natural killer (NK) cells, whereas neutrophil cell counts were first elevated at 24 h after ricin intoxication, and returned later to their basal levels (Figure 7A). This overall massive reduction in cell counts was aptly reflected in the actual weight of the mice spleens. A significant reduction in mean spleen weight was noted at 48 h post-exposure, while at 72 h, the average weight value of the spleens of the ricin-intoxicated mice was half that of the mean value determined in control mice (Figure 7B). Next, we closely quantified cell populations in the BM. In line with the massive cellular atrophy observed in histological sections following intramuscular ricin administration (Figure 3C), we detected a significant reduction in hematopoietic precursors lineage ^negative^ Sca-1^positive^ cKit ^positive^ (LSK) cells, neutrophil, and megakaryocyte cell counts as early as 24 h after intoxication. Reduction of these cell types led to their nearly complete elimination at 48 and 72 h post-exposure. At these time points, nearly complete elimination of B cells was documented as well (Figure 7C). Heart tissue, in addition to the signature parenchymal cardiomyocytes, whose ricin-induced damage was delineated above (Figure 3D), is populated by a large cast of supporter cells, including endothelial cells, fibroblasts, and resident macrophages. A significant decrease in the cardiac endothelial cell population was measured at 48–72 h following ricin administration. No significant changes were found in fibroblast and macrophage counts. However, neutrophils, which are normally absent in this organ, accumulated in the hearts of the ricin-intoxicated mice at 48–72 h after exposure (Figure 7D).

### 2.5. Ricin-Mediated Depurination Activity in Organs of Intoxicated Mice

As ricin related pathology could be a direct outcome of ricin-mediated ribosomal depurination, which leads to protein synthesis termination and cellular death, or an indirect injury stemming from excessive inflammatory and edematous responses [22,23,24], we quantified the catalytic performance of the toxin in various organs. To this end, spleen, BM, heart, liver, kidneys, and lungs of ricin-intoxicated mice were harvested and depurination levels in these organs were calculated. At 24 h following intoxication, low and nearly equal depurination levels (7–9%) were observed in the spleen, BM, and heart, while kidney and lung cells were depurinated to an even lesser extent (4–5%). At this time-point, only the liver exhibited a somewhat higher level of depurination (~13%). The spleen was the soul organ that exhibited depurination levels exceeding 10% at 72 h post-exposure (Table 3). Surprisingly, we found no correlation between ricin catalytic activity measured in various organs and the apparent histopathological severity in these organs. These rather unexpected results seem to suggest that the widespread injuries noted in organs such as the spleen, BM, and heart are mostly of a secondary nature and are not a direct outcome of the catalytic activity of ricin in these organs. 

## 3. Discussion

A key feature of intramuscular ricin exposure is that the toxin, upon entering the blood circulation, can target many organs, rendering therapeutic treatment a challenge. In the present work, we carried out a comprehensive study of the clinical manifestations following intramuscular ricin poisoning in mice. Intramuscular injection of ricin to mice led to hypothermia and hypoglycemia. These signs were previously reported in relation to intraperitoneal and pulmonary ricin exposures [2,25]. Though others have reported that parental injection of ricin via the intravenous or intraperitoneal route induced hypoglycemia that was associated with pathological abnormalities of the pancreas [25,26], we measured only minor amylase elevation with no apparent histological damage in this organ. In addition, intramuscular ricin intoxication caused blood neutrophilia, thrombocytopenia, and induced coagulopathy, the latter being characterized by prolongation of PT and APTT. Tissue focal hemorrhages observed in various organs may indeed stem from this coagulation malfunctioning. Altogether, this set of findings might be interpreted as consumption coagulopathy occurring during the pathophysiologic process of disseminated intravascular coagulation (DIC), however, we were unable to detect any decrease in hemoglobin and erythrocytes, nor any increase in the levels of the fibrin degradation product, D-Dimer, all of which are important features of DIC [27]. In fact, fibrinogen levels were slightly increased, while fibrinogen consumption (decrease) is expected to occur during DIC [27]. It is worthy to note in this context that when ricin enters the circulation, it can bind and destroy the highly susceptible endothelial cells that line the inner side of the blood vessels [21,28]. Platelet adhesion and aggregation can thereby occur at multiple sites of vascular injury, a process that, in turn, would lead to decreased platelet counts in the blood [27]. 

Intramuscular ricin administration induced a formidable proinflammatory response, as manifested by augmented polymorphonuclear cell counts and increased levels of pro-inflammatory cytokines. Unlike in the case of pulmonary exposure to ricin, where elevated levels of IL-6 were measured over long periods of time [1], in the case of intramuscular ricin poisoning, the increase in IL-6 was of a transient nature; high levels of this proinflammatory cytokine were observed only up to 48 h post-exposure. This rather peculiar profile of IL-6 expression might stem from ricin-induced elimination of the cytokine secreting cells, i.e., tissue macrophages that are particularly susceptible to ricin poisoning [29,30]. Histopathological analyses demonstrated local injury of the skin and thigh muscle at the site of ricin injection. Destruction of draining LNs following intramuscular injury of the thigh was autologous to the local necrotic lymphadenopathy determined in Markov’s case [11]. In line with these findings, studies on intramuscularly-administered radio-iodinated ricin in rats demonstrated accumulation of radioactivity in muscle draining LNs [31]. 

Animal studies in which ricin was administered intraperitoneally showed pronounced damage of the liver and kidneys, which may initiate from the early-stage damage of Kupffer cells [32,33]. Following intramuscular ricin intoxication, we observed moderate alterations, including congestion of blood vessels, focal hemorrhages, and necrosis in the liver, kidney, lung, and small intestine. The mild liver and kidney injuries were confirmed also by serum biomarkers, namely detection of slight elevations in the levels of ALP and ALT, as well as BUN and PHOS, assessing liver and renal damage, respectively. Damage to the small intestine in the current ricin-intoxication model was confined to the area of the stem cells in the crypts, with preserved structures of the villi. In contrast, studies in rats demonstrated major pathological changes in the intestine, firstly with infiltration of cells, and then their apoptotic deletion, along with damage to structural cells in the small intestine. This data could be explained by the almost 3-times higher dose of intoxication in the rat model study [34]. 

The widespread ricin-induced damage we observed in the spleen included decreased cellularity and specific reduction in T and B lymphocytes, NK cells, and mononuclear phagocytes. Indeed, a previous study of intravenous distribution of radioactive ricin showed that the highest concentration of radioactivity per tissue weight was observed in the spleen [35]. Further studies documented a profound depletion of splenic lymphocytes following systemic ricin administration [26]. It should be noted that intraperitoneal exposure to ricin also resulted in splenic hypocellularity and atrophy of the white pulp, albeit ricin exposure by the intraperitoneal route entailed megakaryocyte formation. In the intramuscular exposure model presented in the current study, megakaryocyte elimination was striking not only in the spleen, but also in the BM. As platelets are produced mainly from megakaryocytes in the BM and the spleen functions as the platelets’ reservoir, depletion of megakaryocytes in both the BM and the spleen might account for the pronounced thrombocytopenia that we detected. In line with splenic hypocellularity, the BM of the ricin-intoxicated mice also underwent a process of atrophy. Following intoxication, this primary lymphoid organ was devoid of precursor cells, as well as various differentiated cell types. In agreement with our findings, it has been previously demonstrated by others that intravenously injected ricin is able to reach the marrow as efficiently as the liver and kidney [35], though it is not clear whether BM atrophy is an outcome of direct ricin interactions with this tissue, or conversely, a secondary effect. BM suppression can occur due to interruption of the blood supply [36] and if the consequence of BM emptying in the short term is not clear, however, in the long run, suppression of production of erythrocytes, granulocytes, monocytes, lymphocytes, and platelets in the BM would probably have a fatal outcome. 

The effect of ricin on the cardiovascular system has been described in the past; severe myocardial hemorrhages, lowered blood pressure, vasodilation, decreased diastolic compliance, and increased diastolic stiffness were observed in rabbits after intravenous administration of ricin [37,38,39]. A complete atrioventricular conduction block was reported in Markov’s case [11]; ECG abnormalities, including QT interval prolongation, intraventricular conduction disturbance, and repolarization changes were monitored in cases of pediatric castor bean ingestion [40]. In our model, we observed profound myocardial hemorrhages, focal cardiomyocyte destruction or myocyte enlargement in undamaged areas, neutrophilic infiltrates, elevation in cardiac troponin and fibrinogen, and prompt collagen deposition. The latter may lead to distortion of tissue architecture, increased myocardial stiffness, and an inability of the heart to rapidly refill blood for the next contraction [41]. Indeed, in our hands, the main abnormalities of the ECG were hypotension, reduction in R wave amplitude, which reflects depolarization of the main mass of the ventricles, and elevation and widening of a murine distinct J wave at the end of the QRS complex. If in human ECG, the infrequently displayed slight J wave becomes prominent during hypothermia [42]; in mice this reflects the early ventricular repolarization [43]. The elevation of serum potassium, the predominant intracellular cation detected following ricin intoxication, might also play a role in the aberrant cardiovascular conduction and function [44]. As revealed by echocardiography, marked thickening of the walls of the left ventricle and decreased ventricular volume were measured. These alterations were accompanied by a significant reduction in stroke volume and cardiac output, while the ejection fraction was preserved. These formidable cardiac disorders, which in themselves may cause death, are seemingly associated with LV diastolic dysfunctions, such as those observed in Heart Failure with Preserved Ejection Fraction (HFpEF). However, to comply fully with HFpEF, a set of clinical parameters other than diastolic dysfunction, such as exercise intolerance, pulmonary edema, and concentric cardiac hypertrophy, must be documented [45]. In addition, we note that unlike in the reported cases of HFpEF, in our model system no elevation in cardiac macrophage counts was observed [46]. On the other hand, macrophages, which were shown to be the first cell type to succumb during pulmonary ricinosis [6,20,21,22], were not affected in the heart in the case of intramuscular exposure to the toxin. This last finding might indicate that cardiac damage is a secondary effect. In agreement with this hypothesis, our measurements of the direct catalytic performance of the toxin in ricin-intoxicated mice revealed very low amounts of depurinated 28S rRNA in the injured tissues, significantly lower than the ricin-damaged 28S rRNA measured in the lungs, following pulmonary exposure to the toxin. Thus, while levels of depurinated RNA following intranasal exposure of mice to ricin reached up to 40% for total lung cells and 80% for lung epithelial cells [22], depurination levels in the different examined organs following intramuscular exposure to ricin rarely surpassed the 10% threshold. In fact, depurination values determined in the various tested organs at 24–48 h after intramuscular exposure were similar to those acquired as early as 3–6 h in the pulmonary intoxication model. These findings seem to imply that the extensive organ damage observed in the spleen, BM, and heart are not entirely due to the catalytic activity of ricin in these particular organs. 

It should be noted that in the past, we have alluded in two instances to a component of ricin-induced injury, which is not dependent on the catalytic performance of the toxin. In the first case, we monitored the development of ricin-induced toxicity in mice that were previously subjected to whole-body radiation, thereby eliminating their capability to mount an inflammatory response over a substantial period of time. Pre-exposure irradiation did not affect the ricin-mediated depurination activity measured in these mice whatsoever, yet postponed the death of the intoxicated mice significantly, allowing efficient post-exposure treatment over extraordinarily extended periods of time [23]. In a second study, we demonstrated the early disruption of pulmonary tight-junctions following ricin exposure, at such time-points that clearly precede ribosome depurination and cessation of protein synthesis [24]. Taken together with our present results, ricin intoxication appears to be a multi-faceted process, in which indirect disturbances are induced by the toxin in parallel with the toxin’s direct catalytic effect on the cellular protein synthesis system. These indirect ricin-induced pathologies, whose clinical manifestations may differ in a route-of-exposure dependent manner, should be taken into consideration for efficient selection of highly effective medical treatments.

## 4. Materials and Methods 

### 4.1. Animals

Animal experiments were performed in accordance with Israeli law and were approved by the Ethics Committee for animal experiments at the Israel Institute for Biological Research (project identification codes M-01-17, M-38-17, M-76-17, M-34-18, M-35-18, and M-09-19 were approved on January 5, 2017, June 5, 2017, November 22, 2017, July 23, 2018, and January 23, 2019, respectively). Treatment of animals was in accordance with regulations outlined in the USDA Animal Welfare Act and the conditions specified in the National Institute of Health Guide for Care and Use of Laboratory Animals. 

All animals in this study were female CD-1 mice (Charles River Laboratories Ltd., Margate, UK) weighing 27–32 g. Our previous study on intranasal exposure of mice to ricin showed that the lethal dose is similar in mice of the two sexes, which then developed indistinguishable pathological patterns. Based on this a priori knowledge, we performed the current research using one sex only—females—as they are easier to handle throughout the experiments. Prior to exposure, animals were habituated to the experimental animal unit for five days. All mice were housed in filter-top cages in an environmentally controlled room and maintained at 21 ± 2 °C and 55 ± 10% humidity. Lighting was set to mimic a 12/12 h dawn to dusk cycle. Animals had access to food and water ad libitum.

### 4.2. Ricin Preparation and Intoxication

Crude ricin was prepared from seeds of endemic *Ricinus communis*, essentially as described before [47]. Prior to intoxication, mice were anesthetized by an intraperitoneal injection of ketamine (1.9 mg/mouse, Vetoquinol, Lure, France) and xylazine (0.19 mg/mouse, Eurovet Animal Health, AD Bladel, The Netherlands). Crude ricin (1 µL; 18 µg/kg diluted in PBS) was applied intramuscularly into the right thigh and mortality was monitored over 14 d. Preceding these studies, we determined that 9 µg crude ricin/kg body weight is equivalent to 1 mouse intramuscular LD_50_ (95% confidence intervals of 2.96 to 16.4 µg/kg body weight). The mean time to death (MTTD), following intramuscular (i.m.) injection to mice of 18 µg/kg crude ricin (*n* = 35), was determined to be 6.2 ± 0.85 days (Figure S1). 

### 4.3. Biochemical and Hematological Analysis 

For blood sampling, submandibular vein puncture was performed. Glucose concentration was measured using a glucometer (Ascenia Elite XL, Bayer, Leverkusen, Germany, 2015). For differential blood count, blood collected into EDTA containing tubes (Beckton Dickenson, Franklin Lakes, (NJ), USA) was analyzed using the Veterinary Multi-species Hematology System Hemavet 850 (Drew Scientific, Miami Lakes, (FL), USA). For biochemical analysis, blood samples collected in microtainers without coagulant (Beckton Dickenson, USA) were kept at room temperature (RT) for 30 min to allow clotting, and centrifuged at 20,000 g for 1 min for serum separation. Biochemical tests were performed using the VS2 VetScan Chemistry Analyzer (Abaxis, Parsippany, (NJ), USA). Cardiac Troponin I (cTnI) was tested in the serum using a Fluoro-Check^TM^ TRF reader (Nano-Ditech Corporation, city, (state abbr.), USA). For the determination of clotting parameters, blood was collected using sodium citrate (3.2%, Merck-Millipore, Burlington, (Massachusetts), USA) as coagulant. After 30 min incubation in room temperature, samples were centrifuged at 20,000 g for 1 min for plasma separation. PT, APTT, and fibrinogen concentration were determined using TEClot PT, APTT, and Fibrinogen kits on Coatron M2 (TECO, Cranbury, (NJ), USA) according to the manufacturer’s instructions. D-Dimer was determined using Nano-Check^TM^ reader (Nano-Ditech Corporation, USA). 

*Inflammatory mediators.* Tumor necrosis factor-α (TNFα), interleukin-1β (IL-1β), interleukin-6 (IL-6), and tumor growth factor-β1 (TGF- β1) were quantified in the serum using ELISA kits, following the manufacturer’s instructions (R&D Systems, USA).

### 4.4. Histology and Immunohistochemistry

Skin and muscle from hind thigh, ILNs, lungs, liver, kidney, small intestine, pancreas, brain, spleen, bone marrow (BM), and heart were collected and fixed in 4% buffered formaldehyde in PBS pH 7.2–7.4 (Bio Lab, Jerusalem, Israel) for 2 weeks. For decalcification of the BM, bones were washed thoroughly in running water for 30 min and then incubated for 30 h in 8% hydrochloric acid and 8% formic acid (mixed 1:1 ratio). Sections of 5 µm were prepared after paraffin embedding using microtome RM 2255 (Leica, Modiin, Israel) and stained with H&E. For fibrosis visualization, Masson’s Trichrome (Sigma, Rehovot, Israel) staining was performed according to the manufacturer’s instructions. Blue-stained areas (aside of the blue staining of vascular structures) corresponded to fibrotic areas. Analysis were performed using a light microscope Nikon Eclipse E200 (Nikon, Melville, (NY), USA) and images were captured with a coupled camera Digital Sight-Fi1 (Nikon, USA) and analyzed using the NIS-Elements software (Nikon, USA).

For fibrinogen staining, sections were deparaffinized in xylene and rehydrated through ethanol gradient solutions to water. Heat-induced antigen retrieval was performed in Target Retrieval Solution (DAKO) for 30 min at 95 °C. After blocking in 5% BSA in PBS, slides were incubated (overnight, 4 °C) with polyclonal rabbit anti-Fibrinogen (DAKO). Alexa Fluor 594-coupled donkey anti-rabbit antibody was used for detection (Molecular probes^®^, ThermoFisher Scientific, Waltham, (MA), USA). For nuclear staining, slides were mounted with Prolong^®^ Gold antifade reagent containing DAPI (Molecular probes^®^, ThermoFisher Scientific, Carlsbad, CA, USA). Analysis was performed using an LSM 710 confocal scanning microscope (Zeiss, Jena, Germany) equipped with following the lasers: argon multiline (458/488/514 nm), diode 405 nm, DPSS 561 nm and helium-neon 633 nm. Mean fluorescence intensity (MFI) was quantified using the Zen software (version 2.1 Zeiss, Jena, Germany, 2008). 

### 4.5. Permeability Analysis

Heart permeability was determined by the Evans Blue dye (EBD) extravasation method as follows: EBD (7.5 mg/mL, Sigma-Aldrich, Rehovot, Israel) was injected intravenously into mice at a dose of 50 mg/kg, and allowed to circulate for 1 h. Mice were then anesthetized, and the hearts were perfused through the left ventricle with 5 mL PBS. The hearts were removed and EBD was extracted by incubation of the hearts in 0.5 mL of formamide (Sigma-Aldrich, Israel) at 60 °C for 24 h. EBD optical density in the supernatant was measured at 620 nm in a spectrophotometer (Molecular Devices, Sunnyvale, CA, USA) and the total amount of dye was calculated by means of a standard calibration curve. 

### 4.6. Electrocardiogram (ECG) and Echocardiography Recording and Analysis

ECG and echocardiography were performed under 2% isoflurane anesthesia. Mice were placed in a supine position on a heated platform to maintain their body temperature, which was monitored via a rectal probe. To record the ECG signal, three leads were placed subcutaneously in the chest area and hind legs. Recording was performed using BIOPAC System (USA) and analyzed using Acqknowledge software (BIOPAC System, Inc., Goleta, (CA), USA). The onset and offset of P, Q, R, S, J, and T waves were determined according to a previous study [43] and using these time-points, the duration of PQ, QRS, and QRST were estimated. In addition, amplitudes of the P, J, and R waves were defined. Heart rate was calculated as the time between two adjacent R wave picks. Signal-averaged ECGs selected from ~20 stable ECG beats from at least three different time points were analyzed by 2 researchers to confirm the consistency of the data. For echocardiography, the hair from the chest was removed with a depilatory. Acquisition was done in a Vevo 2100 Ultrasound System (VisualSonics Inc.) by 2 researchers. Measurements of left parasternal long and short axes were acquired in B-mode and LV Trace tool enabled calculation of stroke volume, ejection fraction, cardiac output, and fractional shortening. An average of 3–5 measurements were performed for all mice at each time point. 

### 4.7. Flow Cytometry

Perfused hearts were minced into small pieces and subjected to enzymatic digestion with 450 U/mL collagenase I, 125 U/mL collagenase XI, 60 U/mL DNase I, and 60 U/mL hyaluronidase (all Sigma, Israel) for 1 h at 37 °C. Spleens were digested with 1 mg/mL collagenase D (Roche, Mannheim, Germany) for 1 h at 37 °C. BM was flushed out from 2 femurs and 2 tibia. The tissues were then meshed through a 40 µm cell strainer and red blood cells were lysed with red blood cell lysis buffer (Sigma-Aldrich, Israel). For leukocyte staining, cell suspensions were stained with CD45 (clone 30-F11), CD11b (M1/70), CD19 (1D3), CD3 (145-2C11), Ly6G (1A8), CD49b (DX5), Ly6C (HK1.4), F4/80 (BM8), B220 (RA3-6B2), CD11c (N418), MHC class II (M5/114). T and NK cells were identified as CD3^high^ and CD49b^low^, and CD3^low^ and CD49b^high^, respectively. B cells as were defined as C19^high^ or B220^high^ and neutrophils as Ly6G^high^, CD11b^high^. Macrophages were gated as Ly6G^low^, CD11b^high^, F4/80^high^, and dendritic cells as CD11c^high^ MHC class II^high^. For fibroblast (CD45^low^, MEFSK4 ^high^) and endothelial cell (CD45^low^, CD31^high^) staining in the heart, cells were stained with CD31 (390), CD45, and MEFSK4 (clone mEF-SK4). For megakaryocyte staining (CD41^high^), CD41 (MWReg30) followed by goat anti-rat IgG Alexa Fluor 647 (Abcam) were used. For lineage ^negative^ Sca-1^positive^ cKit ^positive^ (LSK) cells, BM cell suspensions were labeled with FITC-conjugated anti-mouse antibodies directed against CD11b, CD49b, CD3, CD4 (Il/41), CD8 (clone), IgM (Il/41), IgD (11-26c), Gr-1 (RB6-8C5) all conjugated to one fluorophore, and Sca-1 (D7) and ckit (2B8). For cardiac Troponin T (cTnT) intracellular staining, heart cells were fixed in 2% formaldehyde, permeabilized with 0.3% saponin, and stained for cTnT (13–11). 

Antibodies were purchased from BioLegend, BD Biosciences, eBioscience, Miltenyi Biotec, or Thermo Fisher Scientific. For dead cell exclusion, 7AAD (eBioscience) was used. Data was acquired on a FACSCalibur (BD Biosciences, San Jose, CA, USA) and analyzed with FlowJo software (version 7.1.2, Tree Star, Ashland, OR, USA, 2007). 

### 4.8. Depurination Assay

Depurination was quantified as previously described [22]. Briefly, a reverse transcriptase (RT) reaction was conducted with two oligonucleotide primers: the first, R-1-GGTAGACACCCTAATACT marked with 6’-fluorescein amidite (FAM), and the second, R-HEX-CTTTGATTGGTCCTAAGGGAGTCATT marked with 5’-hexachloro-fluorescein (HEX). The primers and RNA were incubated with a RT mix containing moloney murine leukemia virus reverse transcriptase (M-MLV RT), dithiothreitol (DTT), deoxynucleotides (dNTPs), and ribonuclease inhibitor (RNasin) (Promega, Madison, WI, USA), for 20 min incubation at 37 °C and then another 20 min at 48 °C. The cDNA that was produced in the reaction was later separated by electrophoresis in the ABI PRISM 310 Genetic Analyzer, using GeneScan application Applied Biosystems (Thermo Fisher Scientific, Waltham, MA, USA). MapMarker 400 X-rhodamine labeled (BioVentures, Wellesley, MA, USA) served as a size marker. 

### 4.9. Statistics

All statistical analyses were conducted with GraphPad Prism software (version 5.01, GraphPad Software Inc., La Jolla, CA, USA, 2007). Data is presented as means ± SEM. For two-group comparisons, two-tailed unpaired Student’s test was used. For multiple comparisons, ANOVA tests followed by Dunnett’s multiple comparison test were applied; *p* < 0.05 were considered significant.

## Figures and Tables

**Table 1:** Biochemical and hematological values in mice following exposure to ricin.

Parameters		Time After Exposure (h)			
		Control	24	48	72
General	Body temp. (±C)	37.7 ± 0.8	37.3 ± 0.8	31.7 ± 2.1 ***	29.5 ± 3.3 ***
	Glucose (mg/dL)	228 ± 48	100 ± 32	74 ± 21 ***	89 ± 33 ***
Serum chemistry	ALP (U/L)	65.4 ± 19	58.8 ± 26	202 ± 228	328.5 ± 7.8 ***
	ALT (U/L)	61 ± 31	235 ± 394	375 ± 284 *	568 ± 485 *
	AMY (U/L)	919 ± 315	719 ± 350	1715 ± 187 ***	2517 ± 2670
	BUN (mg/dL)	17.6 ± 2	22.8 ± 6.7	74.7 ± 54 *	136 ± 39 ***
	PHOS (mg/dL)	9.6 ± 1.8	10.3 ± 1.3	14 ± 0.7 **	16.9 ± 4.9 ***
	Na^+^ (mmol/L)	147 ± 3	145 ± 2	139 ± 2 ***	138 ± 3 ***
	K^+^ (mmol/L)	5.8 ± 1.3	5.5 ± 1	6.1 ± 0.8	7.5 ± 0.6 *
	Cl^−^ (mmol/L)	114 ± 4	112 ± 1	106 ± 2 ***	106 ± 0.6 **
Blood count (K/µL)	WBC	3.7 ± 1.4	4.4 ± 0.7	7.8 ± 3.6 *	8.9 ± 4.6 *
	Neutrophils	1.1 ± 1.4	2.4 ± 0.9 *	6 ± 3.3 **	5.7 ± 3.4 **
	Platelets	617 ± 166	540 ± 240	145 ± 64 ***	113 ± 55 ***
Coagulation	PT (s)	11.35 ± 0.6	11.9 ± 0.8 **	15.4 ± 1.3 ***	17.4 ± 0.4 ***
	APTT (s)	28.5 ± 3	35 ± 8.2 ***	38.4 ± 5.6 ***	35.2 ± 0.7 **
	Fibrin (mg/dL)	235 ± 75	330 ± 110 *	356 ± 71 **	373 ± 8 *
Cytokines (pg/mL)	IL-6	6 ± 0.01	473 ± 200 ***	1404 ± 983 ***	265 ± 124 ***
	TNF-α	0	21 ± 12 **	252 ± 58 ***	252 ± 107 ***
	IL-1β	420 ± 257	529 ± 473	1124 ± 993	1792 ± 1185 *

Body temperature of mice was determined immediately before (control) or at the indicated time points after intramuscular (i.m.) exposure to ricin at a dose of 2x median lethal dose (2LD50, 18 µg ricin/kg body weight). Blood glucose levels, serum chemistry, differential cell counts, coagulation factors, and proinflammatory cytokines were determined in peripheral blood samples collected from mice immediately before (= control) i.m. exposure to ricin or at the indicated time points thereafter. Only parameters that displayed statistically significant changes at 1 or more time-points following ricin intoxication are shown. Data represent mean ± standard error of the mean (SEM); *n* = 4–5 (serum chemistry), *n* = 3 (differential cell counts), *n* = 5–20 (coagulation), *n* = 5–10 (cytokines); * *p* < 0.05, ** *p* < 0.01, *** *p* < 0.001 in comparison to non-intoxicated mice.

**Table 2:** Summary of measured values during ECG analysis.

Parameters	Time After Exposure (h)			
	Control	24	48	72
Heart rate (bpm)	425 ± 70	506 ± 50 **	448 ± 66	357 ± 67 *
PQ interval (ms)	38.7 ± 5	37 ± 4	38.8 ± 8	50.3 ± 15 **
P wave amp. (mV)	0.08 ± 0.03	0.09 ± 0.03	0.09 ± 0.04	0.09 ± 0.02
P segment (ms)	18 ± 4	19 ± 4	23 ± 6 **	29 ± 8 ***
QRS interval (ms)	12.5 ± 1.4	11.7 ± 1.2	12.6 ± 2.2	16 ± 3.7 **
QRST interval (ms)	30.8 ± 8.3	30.6 ± 4.3	45.6 ± 10.9 ***	59.2 ± 16.6 ***
J wave amp. (mV)	0.16 ± 0.05	0.22 ± 0.07 **	0.52 ± 0.16 ***	0.53 ± 0.3 ***
R wave amp. (mV)	0.9 ± 0.2	0.76 ± 0.14 *	0.66 ± 0.16 ***	0.51 ± 1.22 ***

Amplitude (amp.); *n* = 7; * *p* < 0.05, ** *p* < 0.01, *** *p* < 0.001 in comparison to non-intoxicated mice.

**Table 3:** Ricin 28S rRNA depurination values in selected organs of ricin-intoxicated mice.

Time After Exposure (h)	Depurinated 28S rRNA (%)	
	24	48
Spleen	7.3 ± 7.1	15.9 ± 8.5
BM	7.9 ± 3.8	3.7 ± 1.7
Heart	8.9 ± 2.3	4.3 ± 1.5
Liver	13.3 ± 9.7	6.6 ± 3
Kidney	3.9 ± 0.7	9.4 ± 4
Lung	4.8 ± 2.4	7 ± 1.8

Indicated organs, removed from ricin-intoxicated (2LD50, 18 µg ricin/kg body weight) mice at the specified post-exposure time-points, were screened for depurinated 28S rRNA. Value are presented as percentage of ribosome-damaged RNA (depurinated 28S rRNA: total 28S rRNA); *n* = 3–8.

## Supplementary Materials

The following are available online at https://www.mdpi.com/2072-6651/11/6/344/s1, Figure S1: Survival curve of mice following intramuscular (i.m.) ricin administration, Table S1: Biochemical and hematological parameters, which did not change following exposure of mice to ricin.
