# Supplementary material for: Intramuscular Ricin Poisoning of Mice Leads to Widespread Damage in the Heart, Spleen, and Bone Marrow
Source: Toxins (Basel). 2019 Jun 16;11(6):344. doi: 10.3390/toxins11060344 (PMC6628730; doi:10.3390/toxins11060344)
Supplement: Supplementary file 1 [file toxins-11-00344-s001.pdf]

Supplementary Materials: Intramuscular Ricin Poisoning of Mice Leads to Widespread Damage in the Heart, Spleen, and Bone Marrow

Anita Sapoznikov, Amir Rosner, Reut Falach, Yoav Gal, Moshe Aftalion, Yentl Evgy, Ofir Israeli [1], Tamar Sabo and Chanoch Kronman

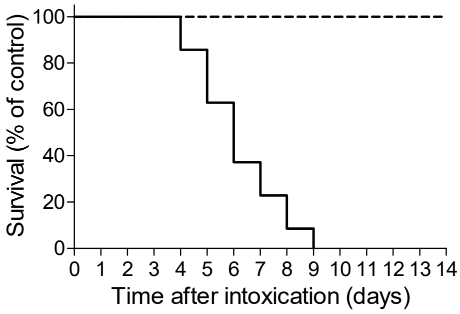

Figure 1. Survival curve of mice following intramuscular (i.m.) ricin administration. Mice were injected i.m. with PBS (dashed line, n = 5) or ricin (2LD$_{50}$, 18 μg ricin/kg body weight, solid line, n = 35) and survival was monitored until day 14 following intoxication.

Table S1. Biochemical and hematological parameters, which did not change following exposure of mice to ricin.

		**Time after exposure (h)**			
		**control**	**24**	**48**	**72**
Serum chemistry	ALB (g/dL)	3.3 ± 0.2	2.9 ± 0.2	1.6 ± 0.85	2.35 ± 0.2
	TBIL (mg/dL)	0.2 ± 0	0.18 ± 0.04	0.3 ± 0.1	0.93 ± 0.95
	CRE (mg/dL)	0.28 ± 0.13	0.24 ± 0.1	0.63 ± 0.21	0.6 ± 0.2
	TP (g/dL)	4.4 ± 0.2	4.2 ± 0.15	4.3 ± 2.6	4.7 ± 1.3
	GLOB (g/dL)	1 ± 0.13	1.28 ± 0.13	1.4 ± 0.5	1.4 ± 1.27
Blood count	LY (K/μl)	2.41 ± 0.9	1.77 ± 0.5	1.56 ± 0.56	2.96 ± 1.27
	MO (K/μl)	0.09 ± 0.04	0.07 ± 0.02	0.09 ± 0.04	0.09 ± 0.09
	EO(K/μl)	0.04 ± 0.03	0.08 ± 0.07	0.12 ± 0.05	0.12 ± 0.1
	BA (K/μl)	0.01 ± 0.01	0.03 ± 0.03	0.02 ± 0.01	0.02 ± 0.02
	HCT (%)	43.6 ± 3.38	48.8 ± 1.84	50.8 ± 5.69	51.7 ± 4.28
	RBC (M/μl)	8.6 ± 0.35	8.9 ± 0.25	10 ± 0.53	10 ± 0.58
	Hb (g/dL)	13.7 ± 0.62	14.3 ± 0.65	16.5 ± 1.3	17 ± 0.55
	MCV (fL)	50.5 ± 2.9	54.7 ± 3.1	50.3 ± 3.6	50 ± 2.8
	MCH (pg)	15.9 ± 0.63	16.1 ± 0.52	16.4 ± 0.67	16.4 ± 0.44
	MCHC (g/dL)	31.5 ± 1.84	29.5 ± 2.2	32.7 ± 2	33 ± 1.8
	MPV (fL)	6.6 ± 0.8	7.5 ± 1.2	8.2 ± 1.3	10.3 ± 0.6
	PDW (%)	44 ± 1.8	43.9 ± 2	54 ± 6.7	45.6 ± 5.4
Coagulation	D-Dimer (ng/ml)	<400	<400	<400	<400
Pro-inflammatory cytokines	TGF-β1 (pg/ml)	ND	ND	ND	ND

Serum chemistry, blood cell counts, coagulation factors and pro-inflammatory cytokines were determined in peripheral blood samples collected from mice immediately before (= control) or at the indicated time points after i.m. exposure to ricin at a dose of 2LD50 (18 µg ricin/kg body weight). ND, not detected. Data represent mean ± STD; n = 4-5 (serum chemistry), n = 3 (blood cell counts), n = 5-20 (coagulation), n = 5-10 (cytokines).
